# Beta-band neural variability reveals age-related dissociations in human working memory maintenance and deletion

**DOI:** 10.1371/journal.pbio.3002784

**Published:** 2024-09-11

**Authors:** Wen Wen, Shrey Grover, Douglas Hazel, Peyton Berning, Frederik Baumgardt, Vighnesh Viswanathan, Olivia Tween, Robert M. G. Reinhart

**Affiliations:** 1 Department of Psychological and Brain Sciences, Boston University, Boston, Massachusetts, United States of America; 2 Tufts University, Department of Biology, Medford, Massachusetts, United States of America; 3 Department of Biomedical Engineering, Boston University, Boston, Massachusetts, United States of America; 4 Center for Systems Neuroscience, Boston University, Boston, Massachusetts, United States of America; 5 Cognitive Neuroimaging Center, Boston University, Boston, Massachusetts, United States of America; 6 Center for Research in Sensory Communication and Emerging Neural Technology, Boston University, Boston, Massachusetts, United States of America; Vanderbilt University, UNITED STATES OF AMERICA

## Abstract

Maintaining and removing information in mind are 2 fundamental cognitive processes that decline sharply with age. Using a combination of beta-band neural oscillations, which have been implicated in the regulation of working memory contents, and cross-trial neural variability, an undervalued property of brain dynamics theorized to govern adaptive cognitive processes, we demonstrate an age-related dissociation between distinct working memory functions—information maintenance and post-response deletion. Load-dependent decreases in beta variability during maintenance predicted memory performance of younger, but not older adults. Surprisingly, the post-response phase emerged as the predictive locus of working memory performance for older adults, with post-response beta variability correlated with memory performance of older, but not younger adults. Single-trial analysis identified post-response beta power elevation as a frequency-specific signature indexing memory deletion. Our findings demonstrate the nuanced interplay between age, beta dynamics, and working memory, offering valuable insights into the neural mechanisms of cognitive decline in agreement with the inhibition deficit theory of aging.

## Abstract 2

Neuronal beta-band oscillations regulate the maintenance and deletion of working memory representations in humans. This study shows that the working memory performance of older adults can be predicted by beta-band neural variability during working memory deletion.

## Introduction

Working memory is a basic cognitive function markedly affected by aging [1,2]. Efficient working memory function is facilitated by multiple processes. On the one hand, processes that promote maintenance of information are important [3]. Emerging research has identified the neural mechanisms contributing to maintenance deficits with age [4]. On the other hand, processes that remove information when it loses its relevance are equally important [5]. Failure to remove irrelevant thoughts from mind can obstruct our capacity-limited systems and interfere with the maintenance of relevant information [6,7]. In fact, a leading theory of neurocognitive aging—the inhibition deficit theory—suggests that impairments in the ability to delete information from working memory are what primarily contribute to age-related decline [8]. Despite the considerable body of work on age-related deletion deficits in distractor inhibition [9], only limited attention has been given to the deletion of targets after responses, with no reference to the underlying neural mechanisms.

Beyond its well-studied role in sensorimotor control, rhythmic neural activity in the beta band (15 to 25 Hz) has been suggested to regulate the status of working memory contents [10–12]. Dynamics in beta-band activity reflect working memory processing. There is a decrease in beta activity when information needs to be maintained and an increase when information needs to be deleted [13]. Maintenance-related beta decrease is primarily observed in the prefrontal cortex [13,14]. By contrast, post-response beta increase is observed among task-related networks involving frontal and centroparietal regions [10], facilitating removal of both memory contents and associated representations such as motor plans after responses. Specifically, neurophysiological evidence from nonhuman primates demonstrated localized post-response beta increase at sites containing memory information during the time course of working memory clear-out [13]. Whether such dynamics can be observed in human electrophysiology and how these neural dynamics change with age is unknown.

We examined the neural mechanisms underlying age-related decline in multiple working memory phases. To accommodate the increased interindividual variability in cognitive aging [2,15], we were further interested in studying neural metrics that are capable of characterizing individual differences in both younger and older adults. Neural variability is an understudied property of brain dynamics, which is increasingly recognized as a sensitive index capable of tracking intra- and interindividual brain–behavior relationships [16–18]. It reflects the joint influence of sensory input, arousal state, attention, and high-order demand variations on brain functions [18]. In particular, behavioral relevance of cross-trial variability has been reported in multiple research fields, with lower variability associated with superior perception [19], more internally guided decision-making [20], and less social conformity behavior [21]. Thus, we leveraged cross-trial neural variability to examine the beta-band oscillatory dynamics during maintenance and after response, with a particular focus on age-related differences.

## Results

### Pronounced age-related working memory deficits with increasing set size

**Fig 1:**
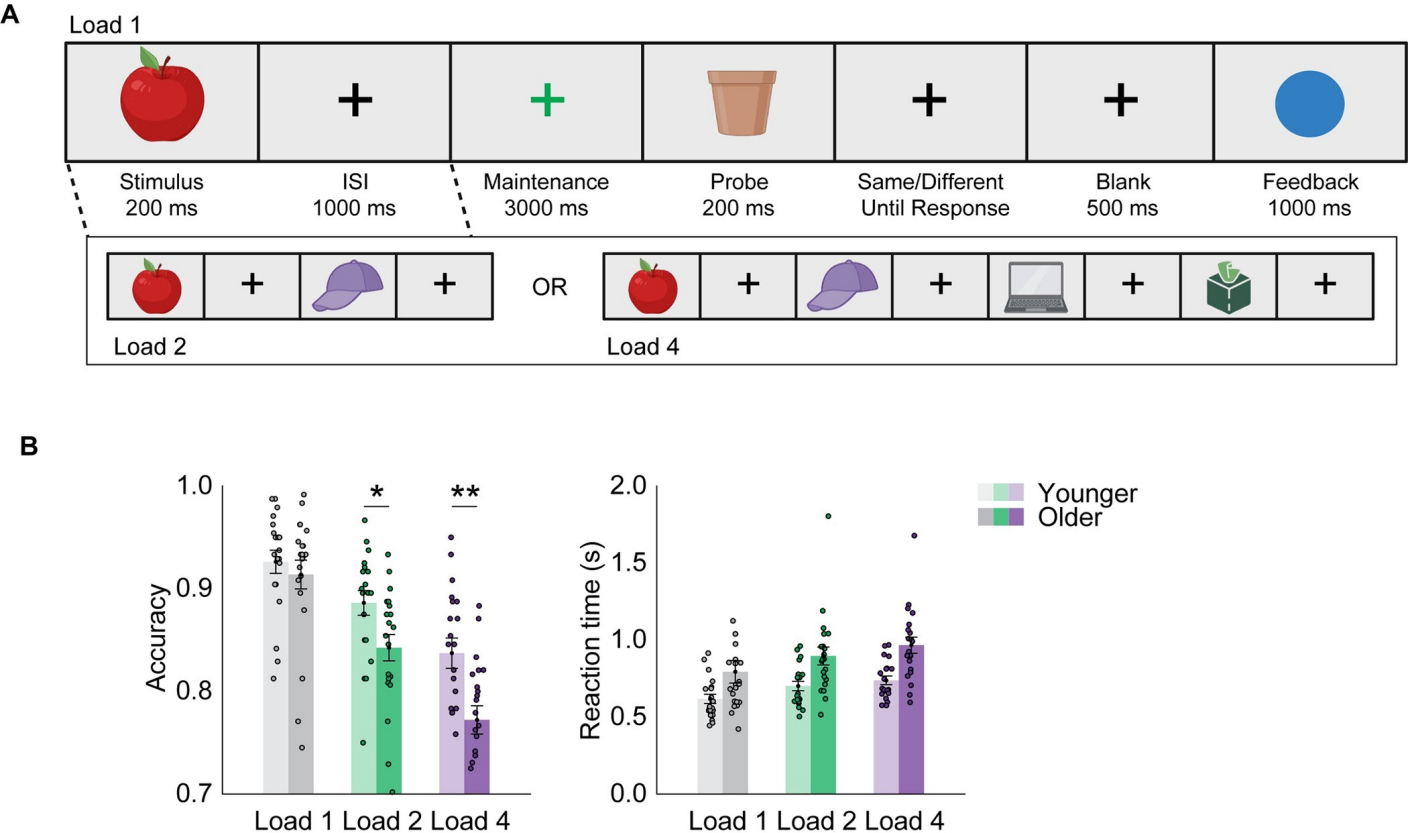
Task and behavioral results. (**A**) Delayed match-to-sample task. One, 2, or 4 images were presented sequentially. Intertrial interval was jittered from a uniform distribution (1.2 to 1.6 s). (**B**) Behavioral results. There is a pronounced age-related memory accuracy decrease at higher loads. No significant age × set size interaction effect was observed in reaction time. Lighter and darker colors represent younger and older adults, respectively. Error bars show standard error of the mean. Circled dots show individual participant data. * *p* < 0.05, ** *p* < 0.01. Source data can be found at https://doi.org/10.5281/zenodo.12735828 (S1 Data).

Twenty younger (22.1 ± 2.5 years) and 21 older (70.6 ± 4.8 years) adults performed a delayed match-to-sample task involving 1, 2, or 4 sequentially presented real-world objects during concurrent electroencephalography (EEG) (**Fig 1A**). Participants were instructed to indicate using a corresponding button press whether a subsequently presented probe item was identical to any one of their memory representations. Feedback was presented 0.5 s after response. Behavioral performance accuracy was better in younger than older participants (**Fig 1B**, F(1, 39) = 5.572, *p* = 0.023, partial χ^2^ = 0.125, BF_10_ = 2.275), at lower compared to higher set sizes (F(2, 78) = 160.548, *p* < 0.001, partial χ^2^ = 0.805, BF_10_ > 100). There was a significant interaction between set size and age group (F(2, 78) = 8.458, *p* < 0.001, partial χ^2^ = 0.178, BF_10_ = 59.334), which was driven by larger age-related working memory deficits at set size 2 and 4 (set size 1, F(1, 39) = 0.47, *p* = 0.497; set size 2, F(1, 39) = 6.18, *p* = 0.017; set size 4, F(1, 39) = 10.39, *p* = 0.003). Reaction time (RT) increased with set size (**Fig 1B**; F(2, 78) = 52.359, *p* < 0.001, partial χ^2^ = 0.573, BF_10_ > 100) and older participants were slower than younger participants (F(1, 39) = 8.788, *p* = 0.005, partial χ^2^ = 0.184, BF_10_ = 7.011). There was no age × set size interaction on RT (F(2, 78) = 1.655, *p* = 0.198, BF_10_ = 0.299).

### Beta variability tracks brain dynamics as hypothesized by cognitive neurophysiological theory of aging

Changes of rhythmic activity at the trial level lead to alterations in trial-averaged power and cross-trial variability (Materials and methods, **S1A Fig**). Rhythmic activity, unless stated otherwise, was measured using cross-trial variability, which captures fluctuations unique to each individual. Cross-trial beta band variability captured the load-dependent neurophysiological changes in younger and older adults predicted by the Compensation-Related Utilization of Neural Circuits Hypothesis (CRUNCH) [22]. CRUNCH posits that older adults would not show a parametric neural change with memory load increases during maintenance. This is because older adults overrecruit resources at low set sizes resulting in a resource shortage when set size further increases. We selected the frontal and centroparietal clusters as the channels of interest due to their relevance to working memory function as revealed in previous studies [23,24].

**Fig 2:**
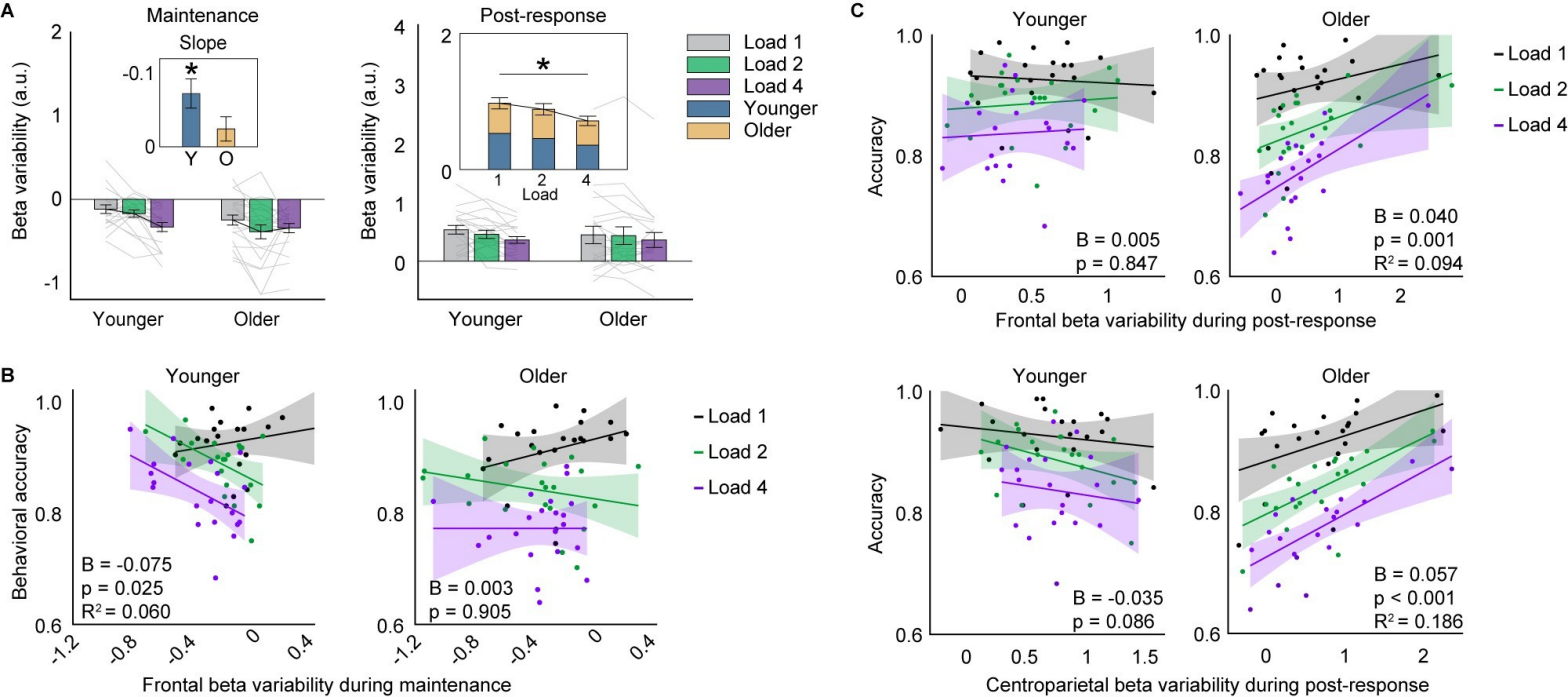
Age-related beta modulation effects during maintenance and post-response. (**A**) Averaged beta-band variability at frontal sites during maintenance (0 to 3 s) and post-response (0.1 to 0.5 s). Inserted panel shows mean slope of load-dependent beta variability during maintenance and main effect of set size during post-response. Gray lines show individual data. (**B**) Maintenance beta variability predicts younger adults’ working memory accuracy. The behavioral relevance of maintenance beta variability was weak at load 1 (Younger: Rho_pearson_ = 0.202, *p* = 0.392; Older: Rho_pearson_ = 0.287, *p* = 0.202), suggesting that maintenance-related activity was not behaviorally predictive when the task was less demanding. (**C**) Post-response beta variability at the frontal and centroparietal clusters predicts older adults’ memory accuracy. For frontal beta variability, there was an outlier in the older group that showed high increase of beta variability. Excluding the outlier did not change the statistical significance of the correlation between post-response variability and memory accuracy (B = 0.055, *p* = 0.004, R^2^ = 0.072). Shaded regions represent 95% confidence intervals. R^2^ represents variance explained by maintenance or post-response beta variability. Source data can be found at https://doi.org/10.5281/zenodo.12735828 (S2 Data).

When examining beta variability of each set size and age group during the maintenance phase, we observed an interaction between age group and set size in the frontal cluster alone (**Fig 2A**, F(2, 117) = 3.886, p = 0.023, partial χ^2^ = 0.087, BF_10_ = 40.957; see **S1 Table** for centroparietal cluster). This suggests that age differentially influences how beta variability changes with memory load. To further quantify this critical interaction effect, we performed linear regression on the beta variability across the 3 set sizes for each participant and tested the slope of their best fit lines at the population level. The parametric variability change with increasing memory load was significant in younger (mean slope = −0.073, t(19) = 3.642, *p* = 0.002, Cohen’s d = 0.814, two-tailed), but not older participants (mean slope = −0.024, t(20) = 1.474, *p* = 0.156). In other words, while there was a load-dependent variability decrease in younger adults, older adults failed to show such a systematic modulation. A closer examination of the beta variability modulation pattern in older adults suggests an inability to further modulate beta variability when the memory load increased from 2 to 4 (t(20) = 0.674, p_bonferroni_ > 1.000), echoing predictions from CRUNCH. Analyses of the encoding phase did not reveal any significant differences between younger and older adults, ruling out the possibility that the observed group differences in load-dependent changes during maintenance stemmed from the encoding phase (**S1 Fig**). Together, these analyses suggest that beta-band dynamics during maintenance capture the fundamental premises of CRUNCH, and cross-trial beta variability is equipped with the sensitivity for investigating mechanistic differences in memory processes between younger and older adults.

### Beta variability during the maintenance phase predicts working memory performance exclusively for younger adults

Since variability-based measures are deemed superior for detecting interindividual differences [18], we leveraged cross-trial variability to assess more fine-grained differences between age groups. To examine whether beta variability during maintenance predicts individual memory performance and whether such an association presents differently between the age groups, we performed a generalized linear mixed model (GLMM), comparing the effect of frontal beta variability estimated across trials of each set size on memory accuracy between younger and older adults (see Materials and methods). We observed a significant interaction of age group and beta variability on memory performance (F(1, 119) = 4.330, *p* = 0.040, partial χ^2^ = 0.035, BF_10_ = 8.545). This suggests a differential relationship between maintenance beta variability and memory performance in younger and older adults. Further analysis revealed that younger participants with lower variability during maintenance performed better (**Fig 2B**; B = −0.075, *p* = 0.025, R^2^ = 0.060), especially when examining set size 2 and 4 (B = −0.137, *p* = 0.001, R^2^ = 0.218). In contrast, beta variability during maintenance failed to predict memory performance for older adults (B = 0.003, *p* = 0.905). This implies that beta variability during the maintenance phase not only showed load-dependent changes at the population level but also predicted interindividual differences in memory performance selectively for younger adults. Aging, on the other hand, appeared to impede these systematic modulations to an extent that behavioral relevance of interindividual beta variability was no longer evident in older adults. The absence of maintenance-related activity in predicting older adults’ performance suggests that, while maintenance is influenced by aging (as evident in CRUNCH-like observations reported above), it may not be the primary working memory processing component that predicts behavioral differences in older adults at the individual level. In light of this finding, we investigated age-related differences beyond the maintenance phase.

### Beta variability during the post-response phase predicts working memory performance exclusively for older adults

Given that maintenance-related beta variability could not track interindividual differences in older adults, we hypothesized that the post-response phase may capture such differences. This hypothesis was derived from 2 premises. One, the inhibition deficit theory implicates deletion deficits to be the primary driver of age-related memory decline [8]. Two, beta rhythmic dynamics post-response, particularly originating from where memory representations are maintained, have been interpreted as the neurophysiological signal of memory deletion [13,25]. Together, these premises link post-response beta rhythms with working memory deficits in aging. To test this hypothesis, we first examined whether frontal beta variability showed systematic changes with set size post-response (0.1 to 0.5 s). Unlike the maintenance phase where such a systematic change was evident only in younger adults, we found that beta variability significantly reduced with increasing set sizes for both younger and older adults (**Fig 2A**; F(2, 117) = 4.852, *p* = 0.009, partial χ^2^ = 0.133, BF_10_ = 98.128), with no significant difference between groups (F(1, 117) = 0.319, *p* = 0.573, BF_10_ = 1.172) or age × set size interaction effect (F(2, 117) = 0.658, *p* = 0.520, BF_10_ = 1.921). These findings suggest that post-response beta dynamics continue to be associated with working memory function despite aging.

Next, we examined whether post-response beta variability predicted individual memory performance of each set size, using a similar GLMM as was performed for the maintenance phase. We found a surprising reversal of patterns relative to those observed during the maintenance phase. Post-response frontal beta variability correlated with memory performance in older but not younger adults (**Fig 2C**; Younger: B = 0.005, *p* = 0.847; Older: B = 0.040, *p* < 0.001, R^2^ = 0.094; age × beta variability interaction, F(1, 116) = 4.155, *p* = 0.044, partial χ^2^ = 0.034, BF_10_ = 5.972). In other words, while individuals’ memory performance for younger adults was driven primarily by frontal beta variability during maintenance, it was the post-response variability that determined memory performance for older adults. A similar pattern was observed for centroparietal channels (age group × beta variability, F(1,119) = 16.736, *p* < 0.001, partial χ^2^ = 0.127, BF_10_ > 100; Older: B = 0.057, *p* < 0.001, R^2^ = 0.186; Younger: B = −0.035, *p* = 0.086). Given the temporal progression between maintenance and post-response phases, one may consider the individual correlation results in older adults as a later manifestation of a maintenance-related effect, perhaps due to overall slowing of information processing with aging [26]. However, this possibility is ruled out when examining the direction of the association between beta variability and memory performance. While reduced variability during maintenance predicted better performance for younger adults, it was increased variability during the post-response phase that predicted better performance for older adults. The dynamic change of beta variability during maintenance and post-response matches observations from previous research [13]. Our findings suggest that older and younger adults differentially leverage beta dynamics during distinct working memory processes to optimize their memory performance. Regulating beta variability during maintenance benefits younger adults but with aging, and perhaps due to the structural and functional reorganization that accompanies it [27], the neurophysiological locus predicting interindividual working memory differences in older adults shifts to the post-response phase.

**Fig 3:**
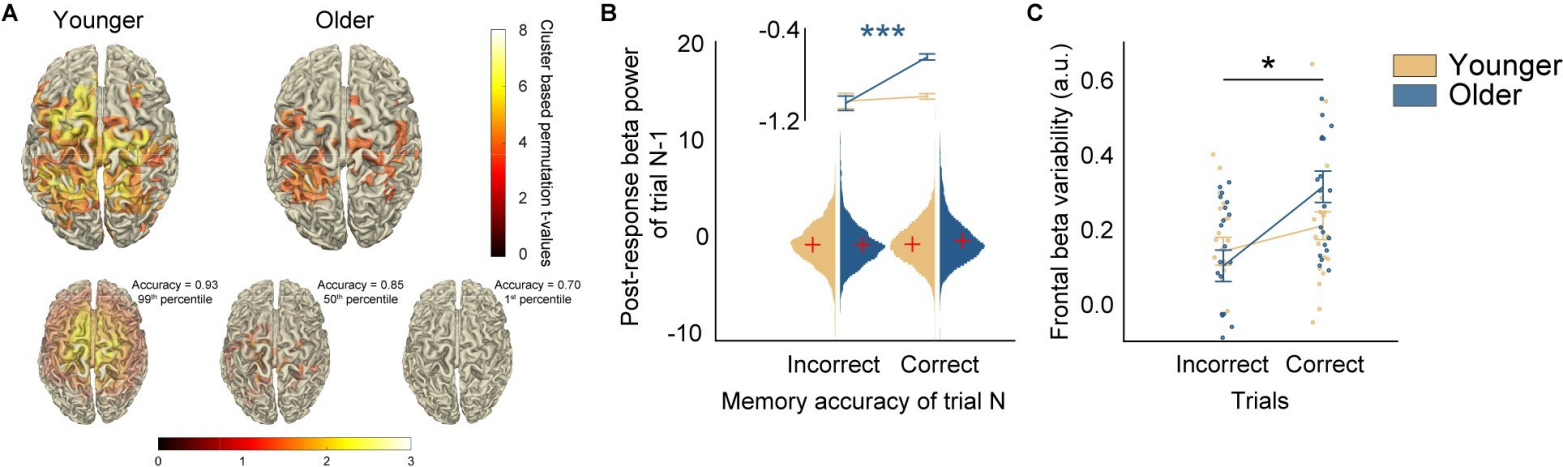
Post-response beta activity. (**A**) Source reconstruction of post-response beta variability. Top panel shows cluster-based permutation *t*-values (*p* < 0.005) when comparing post-response beta increase relative to pre-response (−0.4 to −0.1 s). Exemplars represent older participants in the 99%, 50%, and 1% percentile of the present sample based on averaged memory accuracy. (**B**) Single-trial analysis. Stronger frontal beta activity benefits memory accuracy of the next trial. Violin plot shows the distribution of trial-wise post-response beta power of trial n-1. Red crosses represent the mean. Line plot shows the averaged data. Error bars represent standard error of the mean. (**C**) Correct trials showed larger post-response beta variability increase. Colored dots represent individual participant data. Source data can be found at https://doi.org/10.5281/zenodo.12735828 (S3 Data).

With the post-response phase emerging as the possible locus of memory predictive activity in older adults and previous evidence for marked functional reorganization with aging [27], we examined brain networks recruited during the post-response phase, which may underlie the different contributions of beta variability between younger and older adults. Younger adults, whose memory performance was not associated with post-response beta variability at the individual level, recruited a widely distributed network spanning frontal and centroparietal brain regions (**Figs 3A and S2**) [10]. On the other hand, older adults showed, on average, a spatially restricted network confined to centroparietal regions with a marked absence of frontal engagement. We speculated that the sensitivity of beta variability to individual memory performance in older adults may be related to the extent to which an older individual is able to recruit the frontal cortex during the post-response phase. Indeed, the individual with the best memory performance exhibited pronounced increases in beta variability in frontal regions relative to the participant with median memory accuracy. In contrast, the individual with the lowest memory accuracy did not show any increase in beta variability in any region. While we consider these results qualitative and preliminary, they suggest that the deficient ability to involve the frontal beta rhythms may lead to memory decline with aging. This also contributes to recognizing post-response beta variability as the sensitive index that tracks interindividual differences in older adults.

### Post-response beta dynamics likely index information deletion: Evidence from 2 converging analyses

So far, we have observed a dissociation in the working memory phase where cross-trial variability in the beta band predicts memory performance in younger and older adults.

As previously mentioned, changes in post-response beta activity, indexed by beta bursting or beta power, have been understood as a signature of information deletion [13,28]. Since cross-trial variability is computed using power data from single trials, it is thus possible that cross-trial beta variability also characterizes the deletion process. Indeed, we found power changes at the single-trial level were associated with changes in cross-trial variability (**S1A Fig**), suggesting power and variability changes likely arise from the same cognitive process in this context. To explore this possibility, it is important to verify whether beta activity measured in any form provides collective evidence of information deletion. To this end, we examined how single-trial power, upon which cross-trial variability is computed, impacted memory performance. Then, we extended this analysis and directly examined the association between cross-trial variability and memory performance.

First, we speculated that post-response beta power in each trial should influence the accuracy of the next trial. Specifically, if post-response beta increase indexes deletion of memory representations, then stronger beta power at the single-trial level should benefit performance in the next trial. Consequently, there should be a positive correlation between post-response beta power of the current trial and memory performance of the next trial (brain-to-behavior relation, an “N+1 correlation”). Indeed, larger post-response beta power increase in the previous trial facilitated performance of the current trial exclusively for older adults (**Fig 3B**; Older: t(8832) = 5.662, *p* < 0.001, Cohen’s d = 0.175; Younger: t(9053) = 0.586, *p* = 0.557, permutation *t* test, two-tailed, alpha = 0.001; age groups × post-response beta power of previous trial, F(1, 17817) = 4.296, *p* = 0.038, log odds ratio = 0.458), see **S2 Table** and **S3 Fig**). This pattern of results fits the deletion account, suggesting that a stronger increase in beta power post-response potentially frees up the capacity-limited working memory, thereby benefitting the next trial’s performance.

The preceding analysis rests on power changes in single trials. Does cross-trial variability computed using single-trial power also show evidence in favor of the deletion account? If beta variability also indexes deletion, then the extent to which variability is modulated in the post-response phase might be determined by the strength of the memory representations. When memory representations are weak or partial such that they produce an erroneous response, less deletion will be required on those incorrect trials relative to correct trials. Thus, post-response beta variability should be smaller on incorrect trials relative to correct trials irrespective of age group (behavior-to-brain relation). This was indeed the case. Incorrect trials showed lower frontal beta variability than correct trials (**Fig 3C;** F(1, 38) = 7.052, *p* = 0.012, partial χ^2^ = 0.157, BF_10_ = 3.923). These effects were consistent across both younger and older participants, with no significant difference between age groups (F(1, 38) = 0.615, *p* = 0.438, BF_10_ = 0.451), and no interaction effect of age and correctness (F(1, 38) = 2.106, *p* = 0.155, BF_10_ = 0.794). Thus, post-response frontal beta dynamics were more pronounced in correct trials where the strength of memory representations was stronger and require more deletion.

Both analyses provide converging findings. The behavioral association of single-trial beta power and cross-trial variability fits the predictions of the deletion account of beta rhythms. This provides confidence in our understanding that the specific prediction of interindividual differences in memory performance of older adults during the post-response phase by variability in the beta band likely reflects a facet of the deletion process.

### Control analyses: Ruling out alternative explanations for the post-response beta increase

We have excluded several competing explanations of the post-response beta effect. First, the increase in post-response beta variability should not be interpreted as a reflection of error monitoring. Stronger monitoring is typically reported in incorrect trials compared to correct trials [29]. However, we observed the opposite trend in post-response beta variability, with a more substantial increase found in correct rather than incorrect trials. Moreover, error monitoring signals have typically involved lower frequency activity in the theta band [30], unlike the beta-band frequency under consideration here. As such, beta variability increase is a poor candidate for post-response error monitoring.

Second, increase in post-response beta activity could be interpreted as a feedforward confidence estimation process [31], which plausibly explains the stronger beta variability increase on correct trials relative to incorrect trials, overall. To address this possibility, we again leveraged single-trial beta power upon which variability measures are based. This time, however, we examined the behavioral correlation of single-trial beta power on current trial performance as done in previous studies [31]. Specifically, if beta power indexes confidence, then it should positively correlate with memory performance in the same trial (an N-N correlation). However, this was not the case. Behavioral performance of the current trial could not be explained by post-response beta activity at the trial level (accuracy: F(1, 17877) = 1.276, *p* = 0.259; RT: F(1, 15602) = 0.894, *p* = 0.345), and current trial’s post-response beta power was not modulated by accuracy (F(1, 17877) = 1.044, p = 0.307) or RT (F(1, 15602) = 1.844, *p* = 0.174). Thus, we do not consider confidence-related processing, characterized by post-response beta power increase, to be a compelling explanation in this case. Consequently, we do not consider changes in variability, stemming from power changes, to be reflective of the confidence estimation process either.

Lastly, there is a possibility that the observed N+1 correlation reflects the implementation of a preparatory attentional state rather than deletion of previously held memory representations. However, attentional deployment is typically associated with alpha-band activity [32,33]. Control analyses on post-response alpha activity did not show any significant effects (ps > 0.168). In addition, previous literature suggests that desynchronization of alpha rhythms benefits next trial performance [32]. The direction of this association is opposite in our data where it is enhanced power and variability in the beta band that are reflective of better performance in the following trials. Moreover, given that feedback was presented after responses, preparatory attention in service of the next trial is unlikely to be implemented until feedback offset and the onset of the intertrial interval.

Taken together, we believe that the deletion account of the increased post-response beta power and variability reflects a more coherent and parsimonious explanation than other accompanying cognitive processes during the same information processing window.

### Control analyses: Spectral specificity of the dissociable effects

The dissociable effects we observed as to which memory processing phase contributes to individual performance differences in younger and older adults were specific to the beta band. The critical interaction between age and set size during the maintenance phase was not significant at any other frequency outside the beta band (**S1 Table;** ps > 0.126). We further examined other frequencies in the post-response phase. Again, we did not observe any significant differences between younger and older adults outside the beta band (GLMM age × variability, ps > 0.108). Moreover, the differences we observed during both maintenance and post-response phases could not be explained by signal quality, as there was no significant between-group difference in signal-to-noise ratio (SNR) in any frequency band (ps > 0.101). Thus, differences in how cross-trial variability predicts performance during separate working memory stages in younger versus older adults are spectrally specific to the beta band and cannot be explained by nonspecific changes in the EEG signal.

### Control analyses: Trial-averaged power modulations

Since cross-trial variability is computed using measures of power, and since cross-trial variability results align with those observed using single-trial power data (for instance, N+1 correlations above), we asked whether examining variability provides any additional benefits over and above the examination of conventional trial-averaged power measures alone. It turned out that trial-averaged power failed to capture several significant observations evident through the examination of variability. First, trial-averaged beta power did not show the critical age × set size interaction during maintenance (F(2, 117) = 2.009, *p* = 0.137). Moreover, trial-averaged frontal beta power did not reflect the dissociable interindividual correlation between maintenance-related beta activity and memory performance (Younger: B = −0.022, *p* = 0.316; Older: B = −0.002, *p* = 0.912). Analyses of the trial-averaged post-response spectral activity failed to establish the frequency-specific behavioral relevance of post-response beta activity. Despite the significant correlation between older adults’ memory accuracy and trial-averaged frontal beta power (Older: B = 0.022, *p* < 0.001, R^2^ = 0.117), this nonspecific correlation was also observed in the delta band (Older: B = −0.015, *p* = 0.038, R^2^ = 0.038), alpha band (Older: B = 0.018, *p* = 0.007, R^2^ = 0.062), and gamma band (Older: B = 0.030, *p* = 0.044, R^2^ = 0.036). Post-response beta-band SNR showed no significant difference between age groups (F(1, 39) = 0.042, *p* = 0.838), neither did the trial-averaged beta power (F(1, 39) = 0.052, *p* = 0.820). Of note, the absence of age differences in SNR or trial-averaged power during post-response phase suggests that our results should not be explained by general differences between younger and older adults in the robustness of evoked neural responses to events, which could potentially mark a relevant boundary in a trial. The presence of a significant N+1 correlation using single-trial power together with the absence of age-related maintenance effects and the absence of spectral specificity of post-response beta activity using trial-averaged power imply that trial-wise fluctuations are canceled out in the trial-averaging approach. Thus, cross-trial variability, rather than trial-averaged power, in the beta band, appears to be a trait-like signature that is more sensitive to age-related differences in distinct working memory functions. This agrees with prevailing ideas about the greater sensitivity of variability-based measures in capturing interindividual differences [17–19].

## Discussion

Working memory function is a critical cognitive ability that deteriorates with age following adulthood [34]. But whether different processes within working memory are differentially affected by age remains understudied. This effort is further complicated by the fact that the degree of deterioration is variable across people [2]. Explaining the neurophysiology of working memory decline in aging requires us to examine the constituent processes within working memory simultaneously and consider variability at the interindividual and intraindividual levels as a parameterized function to be explained, rather than mere noise [16]. To this end, we adopted a novel approach to assess between- and within-group differences across ages. We combined cross-trial variability, which has largely been studied with broad-band EEG signal and fMRI hemodynamic responses [16,17,19], with rhythmic dynamics in the beta range, and examined them during both working memory maintenance and post-response deletion phases. Our novel analytical approach suggests that, when considering cross-trial fluctuations of beta power, variability explains individual differences in working memory performance during distinct phases for each age group. Whereas individual memory performance of younger adults was explained by frontal beta variability during maintenance, memory performance of older adults was primarily explained by post-response beta variability. Thus, task-related cross-trial variability augments individual state-dependent characteristics and predicts behavioral differences within and across age groups. With the age-related dissociations between maintenance and post-response phases, beta variability may serve as an age-related, task-sensitive signature of individual differences in distinctive working memory computations.

When developing models of age-related decline in working memory, it is imperative to incorporate the cognitive and neural dynamics during each information processing state. Most theories of aging are coarse-grained at the cognitive level of analysis [26], with little to say regarding distinct information processing phases. As an example, CRUNCH offers a plausible explanation for the pattern of neural effects during maintenance but does not directly address differences in post-response deletion. Our findings provide some relevant insights. For instance, the pattern of results during the post-response stage in the present study suggests impairments in information deletion and a putative inability to recruit compensatory resources. Specifically, CRUNCH would predict the involvement of additional neural processes for rescuing impaired information deletion. This should result in the characteristically saturated neural response pattern with increasing set sizes during the post-response phase, as we observed during the maintenance phase. However, we found consistent positive correlations between post-response beta variability and memory performance across all set sizes. CRUNCH would also predict an overactivation of frontal beta activity or additional engagement of task irrelevant regions during post-response phase to achieve efficient deletion. In contrast, our preliminary source estimation analyses suggest an underrecruitment of frontal regions during the post-response phase. These findings align with the inhibition deficit theory but suggest that compensatory resources, as posited by CRUNCH, could not be instantiated by older adults at least in the present investigation. Perhaps the inability to remove information efficiently during the deletion phase creates a bottleneck. This bottleneck could then influence memory maintenance in the following trial, where compensatory mechanisms during maintenance can still be called upon. In this manner, inefficient information deletion may be one of the reasons for the engagement of compensatory mechanisms during the maintenance phase. By viewing working memory as an information processing system that needs to be continuously regulated, we may be able to bridge the inhibition deficit theory with CRUNCH, through examination of the interdependent nature of information removal and maintenance, as demonstrated in the present study.

We interpret the change in post-response beta dynamics as a reflection of a memory deletion process in agreement with previous studies [13,28]. This interpretation is further supported by the observation of single-trial post-response beta power influencing the memory performance in the next trial. It is possible that changes in single-trial beta power are indices of memory deletion, with cross-trial variability, computed using single-trial power measures, reflecting a trait-like ability to execute and adjust the deletion process when memory needs to be regulated rapidly over trials with varying memory loads. We further think that the overall pattern of results sets the stage for elucidating the nature of the deletion process with greater functional specificity. For instance, it is possible that the increases in post-response beta power and variability signify the demand for deletion (the demand account). This account hypothesizes that stronger beta engagement reflects the absolute amount of information to be deleted. In other words, a stronger increase in trial-wise post-response beta power would reflect a stronger demand for deletion. And since cross-trial variability is computed from, and associated with single-trial power (**S1A Fig**), this relationship may be evident with cross-trial variability also. As a result, this account would expect a larger increase in beta variability with increasing set size. For instance, it is possible that the increase in post-response beta power signifies the demand for deletion. This account hypothesizes that stronger beta engagement (both in terms of single-trial power and cross-trial variability) reflects the absolute amount of information to be deleted. Therefore, this account would expect a larger increase in beta variability with increasing set size. This was not the case in our data where we observed a decrease in post-response beta variability with increasing set size (**Fig 2A**, right). The overall pattern of results can be better explained if we consider beta engagement as a reflection of the efficiency of the deletion process (the efficiency account). The efficiency of deletion may be a composite of the total amount of information to be removed, the state and strength of to-be-deleted memory representations, the time available for the deletion process, and the rate at which information can be deleted. Given a fixed period of time available for uninterrupted deletion (500 ms in the present work), a smaller proportion of information could be removed when the total amount of information to be removed is higher (for instance, load 4) than lower (for instance, load 1). The negative association between beta variability and set size might suggest that a smaller proportion of to-be-removed information has been removed in the window of analysis at higher set sizes compared with lower set sizes. To the extent that information can be removed more efficiently within the same time window, deletion will be facilitated and performance on the next trial will benefit. This explains why we observe the N+1 correlations whereby a stronger beta power in the present trial benefits performance on the following trial, even though, overall, the efficiency of the deletion process may be lower at set size 4 given a limited deletion period. New experiments are needed to test these novel interpretations of beta dynamics. Mapping out the deletion dynamics over post-response periods of different lengths [35] and various memory loads [10] may be a good starting point.

The present findings set the stage for multiple future investigations. For example, it would be prudent to examine the role of object familiarity in the integrity of maintenance and deletion processes. Older people may exhibit some differences in their ability to recall and name objects [36–38], which could at least partially contribute to some memory differences in the present findings. Replicating the study while measuring levels of familiarity and comparing performance with conditions involving abstract, nonnameable objects may be one such approach. It would also help to replicate the findings with larger sample sizes. While the present study was adequately powered to detect an interaction effect between age and set size on memory performance, the interindividual correlations emerging from the present investigation can now be subjected to further follow-up investigations with a larger sample size. In addition, given recent reports suggesting changes in instantaneous beta frequency on a trial-by-trial basis [39], granular investigations on the relationship between deletion and trial-wise or individualized peak frequencies can be implemented. Our findings also hold implications for cognitive processes beyond those being investigated in the present study. For instance, it would be interesting to see whether the beta rhythmic dynamics facilitating deletion in the present study also contribute to other regulatory processes such as deprioritization [40], directed forgetting [41], or controlled removal operations such as information suppression or replacement [6]. Moreover, whether similar neural mechanisms contribute to the transfer of information between working memory and long-term memory needs to be investigated, for it may hold the key to understanding how representations of our continuous experience in working memory are transformed into discrete, segmented representations in long-term memory [42,43]. Finally, future research is needed to test the causal role of beta activity in modulating the influence from deficient deletion to subsequent maintenance in older adults. It may turn out, as our results showing age-related dissociations between maintenance and post-response indicate, that working memory in younger and older adults may have distinct influences of different neural mechanisms in influencing memory performance, suggesting new directions for future model building, and, ultimately, a more comprehensive mechanistic understanding of cognitive aging in health and disease.

## Materials and methods

### Ethics statement

The study protocol was reviewed and approved by the Boston University Institutional Review Board (IRB number 4230E). The research adhered to the ethical guidelines outlined in the Declaration of Helsinki. Written informed consent was obtained from all participants. Participants were compensated $15 per hour.

### Participants

Power analysis (80% power, *p* = 0.05; repeated measures for the critical age × set size interaction) on pilot data (*n* = 10) showed a Cohen’s *f* effect size of 0.248 for the interaction effect, indicating that a total sample size of 28 participants (14 participants per group) would be sufficient to reliably capture an effect. To account for dropouts and exceed these minimum power calculations, we sought at least 20 participants per age group. Twenty-two younger adults and 21 older adults from the greater Boston metropolitan area enrolled in the study. All older participants were prescreened either via phone or an online questionnaire to ensure study eligibility on the following criteria: (1) normal or corrected-to-normal vision and hearing; (2) fluent English speaker; (3) no history of neurological problems or head injury; (4) never been knocked unconscious for longer than 10 minutes; (5) not currently pregnant during the time of study participation; (6) no metal implanted in the head; (7) no implanted electronic devices (pacemaker, neurostimulator); (8) no formal diagnosis of severe tinnitus; (9) no formal diagnosis of a substance problem (related to alcohol or drugs of any kind). Two younger participants’ data were excluded from the analysis due to excessive eye blinks (>60% trials removed in preprocessing). The final sample consisted of 20 younger participants aged 19 to 27 years old (22.1 ± 2.5 years, 10 females, education years 16.0 ± 2.3) and 21 older participants aged 60 to 81 years old (70.6 ± 4.8 years, 7 females, education years 17.3 ± 2.9). Three older participants performed 20 blocks, 23 blocks, and 26 blocks out of a total of 30 blocks due to fatigue.

### Behavioral task

We used object stimuli from a previous study [44]. One, 2, or 4 images of objects were presented sequentially in each trial. Each object was presented for 200 ms followed by an interstimulus interval (ISI) of 1,000 ms. Once all stimuli were presented, the fixation cross turned green. After a delay of 3,000 ms, a probe image was presented for 200 ms, and participants were asked to determine whether the probed image was either identical (50%) or different (50%) from the previous images, by pressing one of 2 buttons on a handheld gamepad. Participants had unlimited time to respond but were instructed to respond as quickly and accurately as possible. Feedback was presented after 500 ms of the response, in the form of a colored circle for 1,000 ms. Yellow indicated a correct response and blue indicated an incorrect response. Color mapping was counterbalanced across participants. New trials began after a jittered time of 1,200 to 1,600 ms (uniform distribution). There were 30 blocks, each containing 24 trials with mixed set sizes, resulting in a total of 720 trials evenly divided among the 3 different set sizes. Participants completed multiple practice blocks until they understood the instruction, showed an averaged accuracy above 0.8, and felt comfortable proceeding.

### Electroencephalography

EEG was recorded at a sampling rate of 1,000 Hz in a dimly lit EEG booth using 64 Ag/AgCl electrodes mounted in a BrainCap elastic cap according to the international 10–20 system. The right mastoid electrode served as the online reference. Data were online bandpass filtered to 0.01 to 125 Hz. Four EOG channels were placed at the outer canthi of each eye, above and below the left eye. Impedance levels were kept below 10 kOhm. Participants were instructed to fixate on the central cross throughout each trial, minimize eye blinks and facial movements, and remain still during each block.

### EEG preprocessing

EEG preprocessing and analysis were conducted using custom Matlab scripts with the EEGLAB [45] and Fieldtrip [46] toolboxes. Raw EEG data were bandpass filtered from 0.5 to 40 Hz and re-referenced to the average of both mastoids. Broken channels were interpolated using a spherical spline method (EEGLAB function, “pop_interp(‘spherical’)”). We extracted epochs time-locked to delay onset (−5.7 to 6 s) and performed independent component analysis (ICA) to correct artifacts caused by eye movements, blinks, heart, muscle, and line noise. The number of removed components was slightly more in older than younger participants (Older: 7.6 ± 2.9; Younger: 6.2 ± 1.6; t(39) = 1.926, *p* = 0.061). We also removed trials with noisy data points that exceeded a voltage threshold of 100 μV. Improbable and abnormally distributed data points beyond 8 standard deviations of the mean probability distribution and kurtosis distribution were also removed. After preprocessing steps, there were 79.4 ±10.2% clean trials from younger adults and 80.0 ± 9.3% from older adults. Additional segmentation was performed depending on time periods of interest with 0 to 3 s for maintenance and 0.1 to 0.5 s post-response phases.

### Behavioral analyses

Mean RT was calculated based on correct trials. Trials with RTs slower than 5 s, or beyond 3 standard deviations of individuals’ own mean RT were excluded from averaging. Two-way mixed Omnibus ANOVA was conducted with the between-participants factor of group (younger versus older) and the within-participants factor of set size (1 versus 2 versus 4). Bonferroni correction was applied for multiple comparisons. Bayesian ANOVA was performed using JASP 0.17.3.

### Time-frequency decomposition

For each trial, we subtracted the averaged waveform of each set size to remove phase-locked activity. Single-trial EEG spectral decomposition was then performed for frequencies ranging from 1 to 40 Hz (1 Hz steps) using Morlet wavelets (width linearly increased from 2 to 10) with a time window of 50 ms. Baseline normalization was performed using decibel conversion relative to the pre-trial (−0.4 to −0.1 s) or pre-response period (−0.4 to −0.1 s) for maintenance and response-locked analysis, respectively. SNR was calculated at the averaged power (signal) divided by the standard deviation of the mean across trials (noise) during the interval 0 to 4 s relative to maintenance onset.

### Variability analysis

The relative variance was calculated as the cross-trial variance change compared to the average of the pre-trial baseline ($\log\left(\frac{Var(S(t))}{mean(Var(baseline))}\right)$). Preplanned analysis of beta variability focused on the frontal cluster defined as Fz and its surrounding channels (AFz, F1, F2, FCz), and the centroparietal cluster defined as CPz and its surrounding channels (Cz, CP1, CP2, Pz). Cross-trial neural variability was calculated for each channel and then averaged within the channel cluster. The averaged relative variance within the frequency band and time interval of interest was statistically compared with the GLMM using Matlab function “fitglme” formulated as “variability ~ age group * set size + (1 | participant)”. Age group and set size were added as fixed effects. Link function was specified as “identity”, which is conventional for normally distributed data. The covariance pattern for the random effect was isotropic. Bayes factor for the GLMM interaction effect was computed by comparing the marginal likelihoods between models with and without the interaction effect.

To examine the load-dependent beta variability changes (**Fig 2A**), we performed linear regression on the beta variability across the 3 set sizes for each participant and tested the slope of their best fit lines versus zero at the population level. To assess the interindividual behavioral relevance of neural variability decrease, while controlling for the intraindividual load-dependent effect during maintenance and post-response (**Fig 2B and 2C**), GLMM was formulated as “averaged memory accuracy ~ age group * beta variability + (1 | set size)”. The intercept of each set size was added as the random effect because interindividual differences rather than intraindividual manipulation (set size effect here) is the primary focus. Moreover, we anticipated that individuals with lower variability during maintenance would show better memory performance across all set sizes. Averaged memory accuracy for each set size would theoretically range from 0 to 1 and the accuracy distribution in our case showed negative skewness. Thus, response distribution was specified as “Gamma” with inverse link function. We further confirmed the result by normalizing the response variable (Matlab function “betafit” and “betainv”). With the normalization, the link function was specified as “identity” and the response distribution was specified as “normal”. Regardless of the choices of link function and response distribution, results were consistent (maintenance: F(1, 119) = 3.806, *p* = 0.053, BF_10_ = 6.473; post-response: F(1, 116) = 3.681, *p* = 0.058, BF_10_ = 6.102). Regarding the significant interaction term between age group and beta variability, we constructed a regression model that included set size and beta variability as predictors separately for each age group. Similar analyses were performed on post-response frontal beta variability. An outlier in the older group showing a large post-response frontal beta increase (**Fig 2C**) was identified and excluded from the ANOVA analysis.

Comparison between correct and incorrect trials was performed on load-4 trials to obtain a sufficient number of incorrect trials (**Fig 3C**). Given the unbalanced proportion of correct and incorrect trials, we subsampled from correct trials to avoid bias arising from the unequal number of trials and iterated for 200 times. The averaged results of all iterations were used for statistical tests. An outlier was excluded from the Omnibus ANOVA analysis. Including the outlier did not alter the conclusion (F(1, 39) = 6.177, *p* = 0.017, partial χ^2^ = 0.137, BF_10_ = 3.016).

### Single-trial analysis

N+1 analysis was performed to reveal the behavioral influence of post-response beta activity on the next trial. Since variability is an aggregated index across trials, we used trial-wise power for this analysis. Single-trial beta power during maintenance (0 to 3 s after stimulus onset) and post-response (0.1 to 0.5 s after response, with the first 100 ms removed to avoid motor artifacts and temporal smearing) were added into the GLMM model with the formula “Memory accuracy (n) ~ maintenance beta power (n) * set size (n) * post-response beta power (n-1) * set size (n-1) * age group + (1 + maintenance beta power + post-response beta power (n-1) + set size (n) + set size (n-1) | participant)”. The response distribution was specified as “Binomial” and the link function was specified as “logit”. We hypothesized that memory accuracy of the current trial was determined by set size and maintenance activity of the current trial, set size of the previous trial as well as the extent to which previous information was deleted. Participants’ intercept and slope variations of the fixed effect were added as the random term. Given the significant interaction effect between age group and post-response beta power of trial n-1, we separated trial n based on response accuracy and compared the post-response beta power of trial n-1 between correct and incorrect trials in each age group using a permutation *t* test (**Fig 3B**, permutation times = 10,000, two-tailed, alpha = 0.001). The same analysis was conducted on within-trial variability calculated as the variance of normalized beta power during the maintenance and post-response phases.

N-N analysis was performed to investigate the relationship between post-response beta power and response confidence at the trial level. We examined whether the post-response beta power of the current trial could explain the same trial’s behavioral performance (“accuracy (n) or RT (n) ~ post-response beta power (n) * group * set size (n) + (1+ post-response beta power + set size (n) | participant)”). When using RT as the response index, the link function was specified as “inverse” for the gamma distribution. Only correct trials were included for the analysis of RT. When using accuracy as the response index, the link function was specified as “logit” for the binomial distribution. Additionally, we modeled the influence of response on post-response beta power (“post-response beta power (n) ~ RT (n) or accuracy (n) * group * set size (n) + (1+set size (n) + RT (n) or accuracy (n) | participant)”) with the link function specified as “identity” for the normal distribution.

### Source localization

Linearly constrained minimum variance (LCMV) beamforming was used to reconstruct the cortical sources of post-response neural variability changes. Sensor-level data were referenced to the common average. A standard anatomical MRI and a boundary element method (BEM) headmodel from the Fieldtrip toolbox were used to construct a 3D template grid at 1 cm resolution in Montreal Neurological Institute template space. Given the distance between EEG electrodes and the scalp, we moved the brain surface 5 mm inwards from the skull to accommodate BEM stability (“ft_prepare_sourcemodel”). Channel neighborhood was defined using the default EEG template (“easycapM11_neighb.mat”). A common spatial filter was computed based on band-passed (15 to 25 Hz) EEG time series of all set sizes. Virtual channel time courses for all voxels were reconstructed separately for each set size using the common filter. We then performed time-frequency decomposition and calculated the relative variance on this virtual-channel data as we did on the sensor level. The leadfield comprises 4,050 grids. Source space cluster-based permutation tests were conducted using paired *t* tests on response relative to the pre-response baseline (−0.4 to −0.1 s). Monte Carlo nonparametric randomization was iterated for 10,000 times with the alpha level of the permutation test set to 0.005.

## Supporting information

S1 Fig Beta variability during encoding. Related to Fig 2. (**A**) Example data for load 1. Gray curves represent trial-wise beta power. The purple curve represents the average of all trials, and the green curve illustrates cross-trial variability. Cross-trial beta variability and trial-averaged beta power showed consistent patterns across time. They were correlated during maintenance (Load 1: Pearson’s Rho = 0. 841, *p* < 0.001; Load 2, Pearson’s Rho = 0.871, *p* < 0.001; Load 4, Pearson’s Rho = 0.683, *p* < 0.001) and post-response phases (Load 1: Pearson’s Rho = 0. 939, *p* < 0.001; Load 2, Pearson’s Rho = 0.951, *p* < 0.001; Load 4, Pearson’s Rho = 0.947, *p* < 0.001). Changes in single-trial beta power affect cross-trial variability. When we median-split beta power of each participant and compared the variance between subset of trials with higher and lower beta power, we found that trials with higher beta power had greater variance than those with lower beta power (maintenance: t(40) = 3.754, *p* < 0.001, Cohen’s d = 0.747; post-response: t(40) = 1.818, *p* = 0.038, Cohen’s d = 0.275, one-tailed *t* test). (**B**) Beta-band variability time course. Vertical dashed lines denote stimulus onsets. (**C**) Item-specific variability during encoding. Data were averaged from variability changes during each stimulus presentation (0 to 1.2 s). (**D**) Slope of variability changes. Boxplot shows the distribution of slopes. Slopes were obtained from linear fitting of beta variability changes induced by consecutive stimulus presentations in both load 2 and load 4. The central line represents the median, and black crosses represent outlier data points beyond 1.5 times the interquartile range. No significant age-related differences were observed in the slopes (Load 2: t(39) = 1.054, *p* = 0.298; Load 4: t(39) = 1.052, *p* = 0.299). Source data can be found at https://doi.org/10.5281/zenodo.12735828 (S4 Data).
(TIF)

S2 Fig Cross-trial neural variability during maintenance and post-response. Related to Figs 2 and 3. (**A**) Time-frequency map of neural variability at frontal sites. (**B**) Frontal beta variability during maintenance phase. Time frequency (difference) map shows frontal neural variability averaged across set sizes. Topographical plots show the averaged beta-band variability (15 to 25 Hz) during the maintenance interval (0 to 3 s). Black dots on the topography highlight the frontal channels used to generate time-frequency maps. Shaded error bars on the time series represent the between-participant standard error. The colored vertical solid lines on the x-axis correspond to the mean RT of each set size. (**C**) Time-frequency (difference) map of post-response neural variability changes at the frontal site. (**D**) Topography of post-response (0.1 to 0.5 s) beta variability increases. The highlighted channels passed cluster-based permutation tests (alpha = 0.001, two-tailed).
(TIF)

S3 Fig Related to Fig 3. The influence of post-response beta power of previous trials on memory accuracy. (**A**) The interaction effect among set size of the previous trial, post-response beta power of the previous trial, and the current trial’s set size. (**B**) The interaction effect of set size of previous trials, post-response beta power of the previous trial, and age groups. (**C**) The interaction effect among set size of previous trials, post-response beta power of the previous trial, current trial’s set size, and age groups. Error bars represent standard error of the mean. Source data can be found at https://doi.org/10.5281/zenodo.12735828 (S3 and S5 Data).
(TIF)

S1 Table Related to Fig 2. Analysis of maintenance-related activity. The critical interaction between age group and set size was observed in the beta band (15–25 Hz). The frequency band of interest was guided by existing literature; however, we also explored other frequency bands to demonstrate frequency specificity. While other spatiospectral combinations did not show significant age × set size interaction effects, we proceeded to separate age groups and examined interindividual correlation between maintenance activity and memory accuracy for completeness.
(DOCX)

S2 Table Related to Fig 3. GLMM output table of single-trial N+1 analysis using the formula “Accuracy (n) ~ set size (n) * maintenance beta power (n) * set size (n-1) * post-response beta power (n-1) * age group+ (1 + set size (n) + maintenance beta power (n) + set size (n-1) + post-response beta power (n-1) | participant)”. Participants’ accuracy of the current trial (n) is determined by set size and maintenance beta power of the current trial n, as well as set size and post-response beta power of the previous trial n-1.
(DOCX)

S1 Data Source data of Fig 1. (CSV)

S2 Data Source data of Fig 2. (XLSX)

S3 Data Source data of Fig 3. (XLSX)

S4 Data Source data of S1 Fig. (XLSX)

S5 Data Source data of S3 Fig. (XLSX)

## Abbreviations

BEM: boundary element method
CRUNCH: Compensation-Related Utilization of Neural Circuits Hypothesis
EEG: electroencephalography
GLMM: generalized linear mixed model
ICA: independent component analysis
ISI: interstimulus interval
LCMV: linearly constrained minimum variance
RT: reaction time
SNR: signal-to-noise ratio
