# Supplementary figures and images for: Beta-band neural variability reveals age-related dissociations in human working memory maintenance and deletion
Source: PLoS Biol. 2024 Sep 11;22(9):e3002784. doi: 10.1371/journal.pbio.3002784 (PMC11389900; doi:10.1371/journal.pbio.3002784)

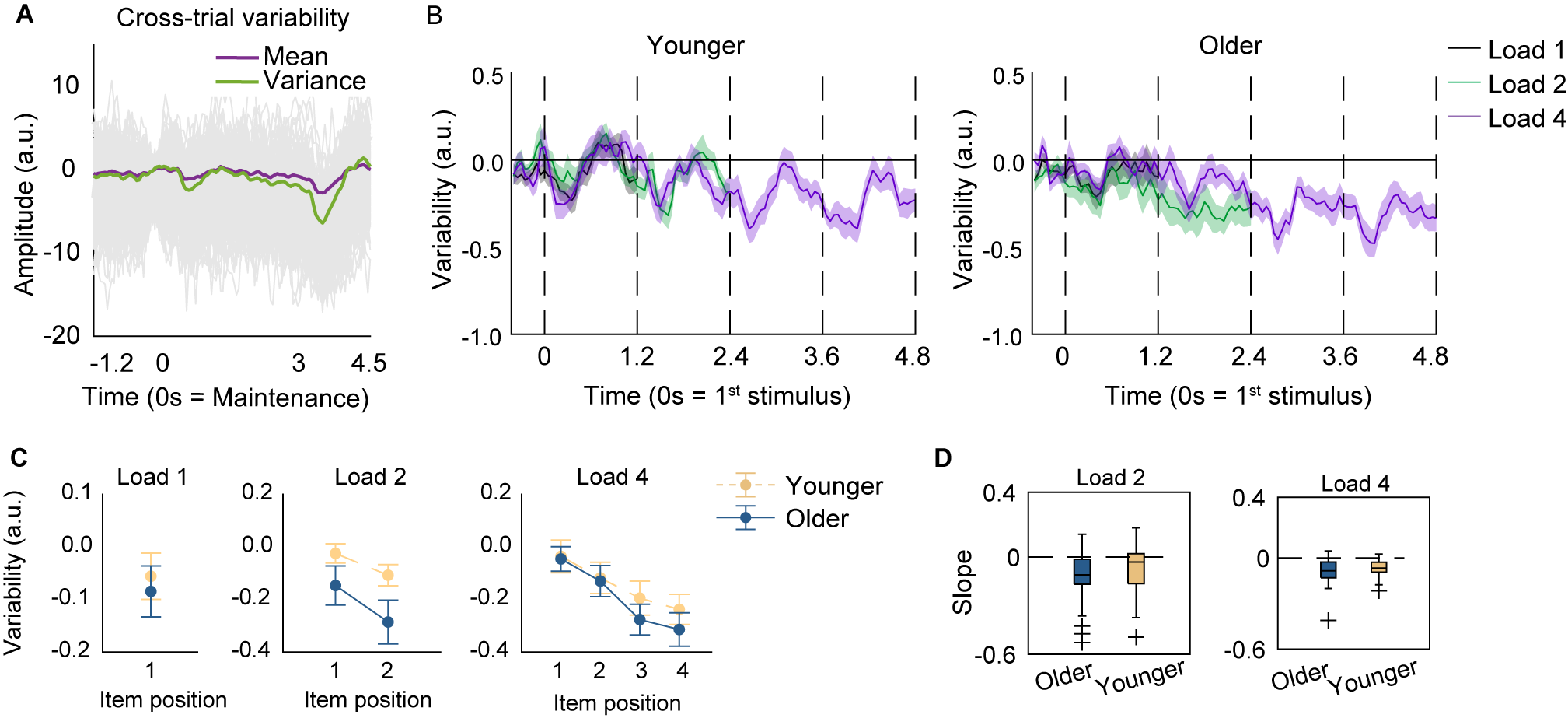

Supplement: S1 Fig — Related to Fig 2. (A) Example data for load 1. Gray curves represent trial-wise beta power. The purple curve represents the average of all trials, and the green curve illustrates cross-trial variability. Cross-trial beta variability and trial-averaged beta power showed consistent patterns across time. They were correlated during maintenance (Load 1: Pearson’s Rho = 0. 841, p < 0.001; Load 2, Pearson’s Rho = 0.871, p < 0.001; Load 4, Pearson’s Rho = 0.683, p < 0.001) and post-response phases (Load 1: Pearson’s Rho = 0. 939, p < 0.001; Load 2, Pearson’s Rho = 0.951, p < 0.001; Load 4, Pearson’s Rho = 0.947, p < 0.001). Changes in single-trial beta power affect cross-trial variability. When we median-split beta power of each participant and compared the variance between subset of trials with higher and lower beta power, we found that trials with higher beta power had greater variance than those with lower beta power (maintenance: t(40) = 3.754, p < 0.001, Cohen’s d = 0.747; post-response: t(40) = 1.818, p = 0.038, Cohen’s d = 0.275, one-tailed t test). (B) Beta-band variability time course. Vertical dashed lines denote stimulus onsets. (C) Item-specific variability during encoding. Data were averaged from variability changes during each stimulus presentation (0 to 1.2 s). (D) Slope of variability changes. Boxplot shows the distribution of slopes. Slopes were obtained from linear fitting of beta variability changes induced by consecutive stimulus presentations in both load 2 and load 4. The central line represents the median, and black crosses represent outlier data points beyond 1.5 times the interquartile range. No significant age-related differences were observed in the slopes (Load 2: t(39) = 1.054, p = 0.298; Load 4: t(39) = 1.052, p = 0.299). Source data can be found at https://doi.org/10.5281/zenodo.12735828 (S4 Data). (TIF) [file pbio.3002784.s001.tif]

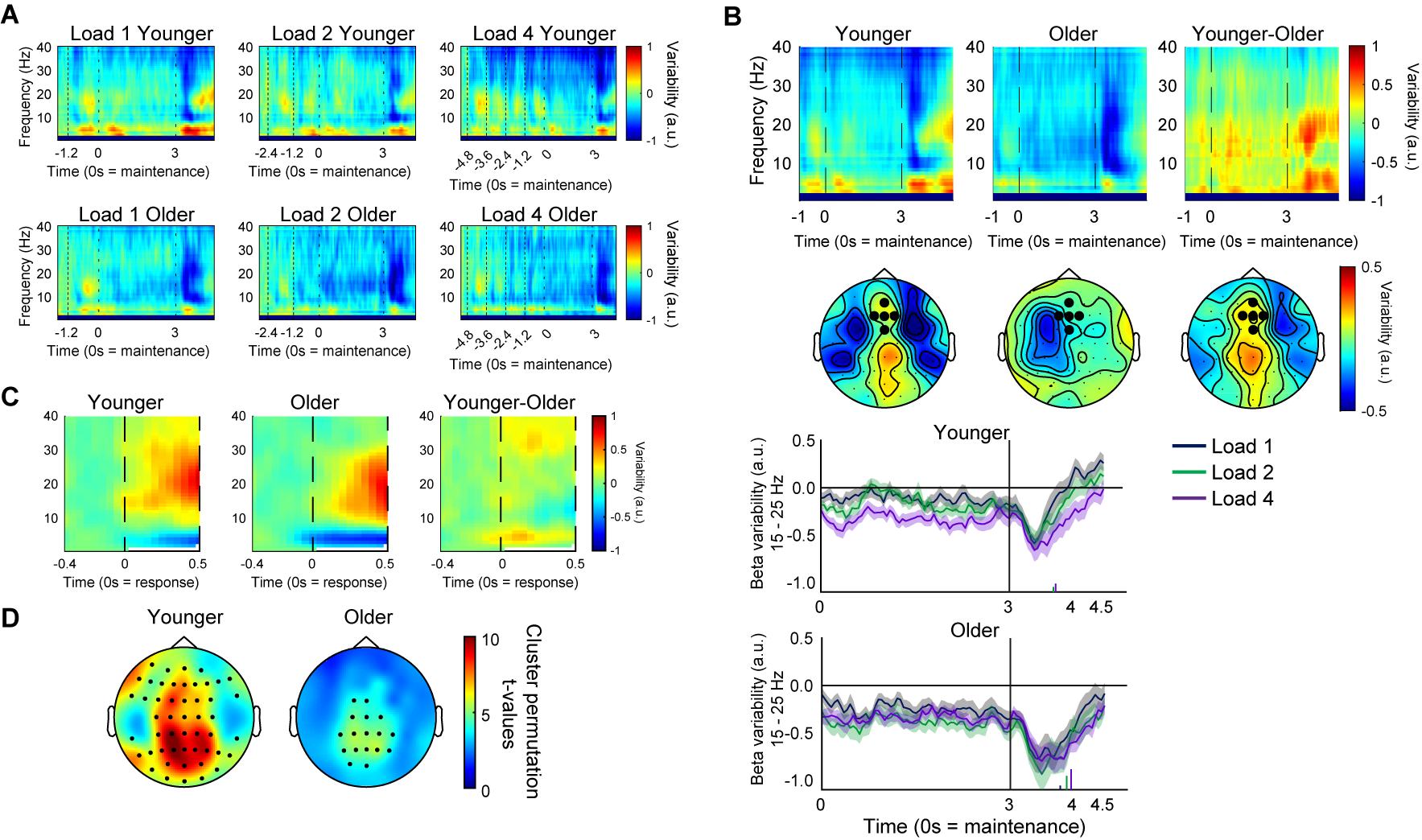

Supplement: S2 Fig — Related to Figs 2 and 3. (A) Time-frequency map of neural variability at frontal sites. (B) Frontal beta variability during maintenance phase. Time frequency (difference) map shows frontal neural variability averaged across set sizes. Topographical plots show the averaged beta-band variability (15 to 25 Hz) during the maintenance interval (0 to 3 s). Black dots on the topography highlight the frontal channels used to generate time-frequency maps. Shaded error bars on the time series represent the between-participant standard error. The colored vertical solid lines on the x-axis correspond to the mean RT of each set size. (C) Time-frequency (difference) map of post-response neural variability changes at the frontal site. (D) Topography of post-response (0.1 to 0.5 s) beta variability increases. The highlighted channels passed cluster-based permutation tests (alpha = 0.001, two-tailed). (TIF) [file pbio.3002784.s002.tif]

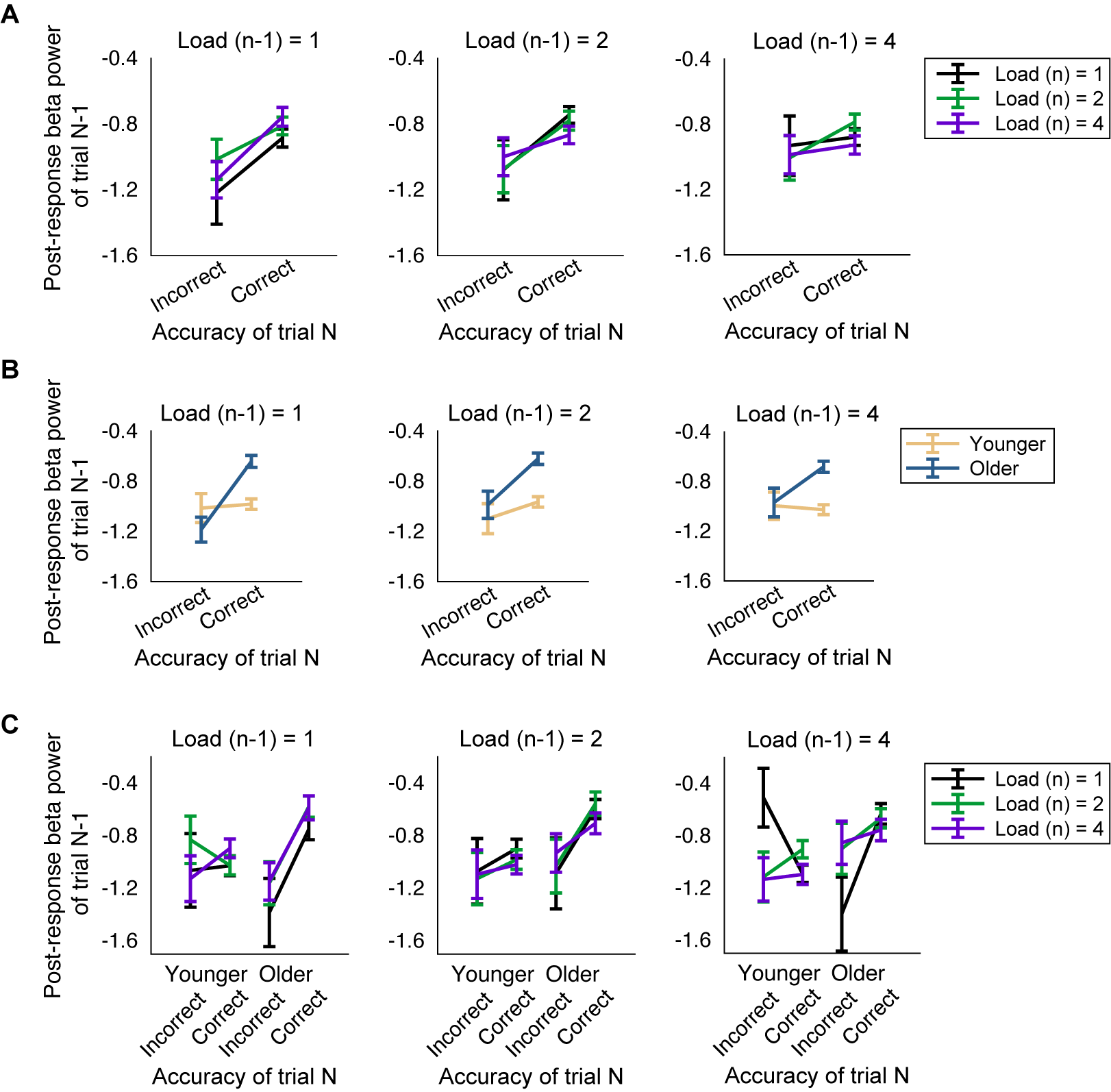

Supplement: S3 Fig — The influence of post-response beta power of previous trials on memory accuracy. (A) The interaction effect among set size of the previous trial, post-response beta power of the previous trial, and the current trial’s set size. (B) The interaction effect of set size of previous trials, post-response beta power of the previous trial, and age groups. (C) The interaction effect among set size of previous trials, post-response beta power of the previous trial, current trial’s set size, and age groups. Error bars represent standard error of the mean. Source data can be found at https://doi.org/10.5281/zenodo.12735828 (S3 and S5 Data). (TIF) [file pbio.3002784.s003.tif]
