# Supplementary material for: Beta-band neural variability reveals age-related dissociations in human working memory maintenance and deletion
Source: PLoS Biol. 2024 Sep 11;22(9):e3002784. doi: 10.1371/journal.pbio.3002784 (PMC11389900; doi:10.1371/journal.pbio.3002784)
Supplement: S1 Table — Analysis of maintenance-related activity. The critical interaction between age group and set size was observed in the beta band (15–25 Hz). The frequency band of interest was guided by existing literature; however, we also explored other frequency bands to demonstrate frequency specificity. While other spatiospectral combinations did not show significant age × set size interaction effects, we proceeded to separate age groups and examined interindividual correlation between maintenance activity and memory accuracy for completeness. (DOCX) [file pbio.3002784.s004.docx]

**Supporting Information for**

**Beta-band neural variability reveals age-related dissociations in human working memory maintenance and deletion**

Wen Wen ^1^, Shrey Grover ^1^, Douglas Hazel ^6^, Peyton Berning ^1^, Frederik Baumgardt ^1^, Vighnesh Viswanathan ^1^, Olivia Tween ^1^, Robert M. G. Reinhart ^1-5^

Correspondence to:

Robert M. G. Reinhart

rmgr@bu.edu

**Table S1. Analysis of maintenance-related activity**. The critical interaction between age group and set size was observed in the beta band (15-25 Hz). The frequency band of interest was guided by existing literature, however, we also explored other frequency bands to demonstrate frequency specificity. While other spatiospectral combinations did not show significant age x set size interaction effects, we proceeded to separate age groups and examined inter-individual correlation between maintenance activity and memory accuracy for completeness.

| Frequency | Channel cluster | GLMM ANOVA (AgeGroup*SetSize) | Younger | Older |
| --- | --- | --- | --- | --- |
| 1-3Hz | Frontal | Interaction: F(2, 117) = 0.184, p = 0.832 Group: F(1, 117) = 1.486, p = 0.225 Set size: F(2, 117) = 0.649, p = 0.524 | B = -0.006, p = 0.702 | B = 0.013, p = 0.370 |
| 4-7Hz | Frontal | Interaction: F(2, 117) = 0.403, p = 0.669 Group: F(1, 117) = 3.876, p = 0.051 Set size: F(2, 117) = 2.961, p = 0.056 | B = -0.003, p = 0.890 | B = -0.005, p = 0.792 |
| 8-12Hz | Frontal | Interaction: F(2, 117) = 0.900, p = 0.409 Group: F(1, 117) = 2.622, p = 0.109 Set size: F(2, 117) = 1.428, p = 0.243 | B = 0.015, p = 0.419 | B = -0.025, p = 0.283 |
| 15-25Hz | Frontal | Interaction: F(2, 117) = 3.886, p = 0.023 Group: F(1, 117) = 2.483, p = 0.118 Set size: F(2, 117) = 8.820, p < 0.001 | B = -0.075, p = 0.025 | B = 0.003, p = 0.905 |
| 28-40Hz | Frontal | Interaction: F(2, 117) = 2.106, p = 0.126 Group: F(1, 117) = 0.095, p = 0.759 Set size: F(2, 117) = 4.594, p = 0.012 | B = -0.041, p < 0.001 | B = 0.029, p = 0.104 |
| 1-3Hz | Centroparietal | Interaction: F(2, 117) = 2.381, p = 0.097 Group: F(1, 117) = 0.169, p = 0.681 Set size: F(2, 117) = 2.009, p = 0.139 | B = -0.013, p = 0.381 | B = 0.017, p = 0.174 |
| 4-7Hz | Centroparietal | Interaction: F(2, 117) = 0.763, p = 0.469 Group: F(1, 117) = 3.094, p = 0.081 Set size: F(2, 117) = 0.223, p = 0.801 | B = 0.000, p = 0.993 | B = 0.031, p = 0.033 |
| 8-12Hz | Centroparietal | Interaction: F(2, 117) = 2.756, p = 0.068 Group: F(1, 117) = 8.788, p = 0.004 Set size: F(2, 117) = 1.286, p = 0.280 | B = 0.019, p = 0.331 | B = 0.017, p = 0.302 |
| 15-25Hz | Centroparietal | Interaction: F(2, 117) = 0.180, p = 0.836 Group: F(1, 117) = 6.263, p = 0.014 Set size: F(2, 117) = 2.001, p = 0.140 | B = 0.049, p = 0.139 | B = 0.025, p = 0.458 |
| 28-40Hz | Centroparietal | Interaction: F(2, 117) = 0.938, p = 0.394 Group: F(1, 117) = 0.020, p = 0.889 Set size: F(2, 117) = 4.337, p = 0.015 | B = -0.042, p = 0.008 | B = 0.059, p = 0.057 |
