# Supplementary material for: Beta-band neural variability reveals age-related dissociations in human working memory maintenance and deletion
Source: PLoS Biol. 2024 Sep 11;22(9):e3002784. doi: 10.1371/journal.pbio.3002784 (PMC11389900; doi:10.1371/journal.pbio.3002784)
Supplement: S2 Table — GLMM output table of single-trial N+1 analysis using the formula “Accuracy (n) ~ set size (n) * maintenance beta power (n) * set size (n-1) * post-response beta power (n-1) * age group+ (1 + set size (n) + maintenance beta power (n) + set size (n-1) + post-response beta power (n-1) | participant)”. Participants’ accuracy of the current trial (n) is determined by set size and maintenance beta power of the current trial n, as well as set size and post-response beta power of the previous trial n-1. (DOCX) [file pbio.3002784.s005.docx]

**Supporting Information for**

**Beta-band neural variability reveals age-related dissociations in human working memory maintenance and deletion**

Wen Wen ^1^, Shrey Grover ^1^, Douglas Hazel ^6^, Peyton Berning ^1^, Frederik Baumgardt ^1^, Vighnesh Viswanathan ^1^, Olivia Tween ^1^, Robert M. G. Reinhart ^1-5^

Correspondence to:

Robert M. G. Reinhart

rmgr@bu.edu

**Table S2. GLMM output table of single trial N+1 analysis.** The formula is ‘Accuracy (n) ~ set size (n) * maintenance beta power (n) * set size (n-1) * post-response beta power (n-1) * age group + (1 + set size (n) + maintenance beta power (n) + set size (n-1) + post-response beta power (n-1) | participant)’. Participants’ accuracy of the current trial (n) is determined by set size and maintenance beta power of the current trial n, as well as set size and post-response beta power of the previous trial n-1.

| Term | FStat | DF1 | DF2 | pValue |
| --- | --- | --- | --- | --- |
| (Intercept) | 30.812 | 1 | 17817 | 0.000 |
| set size (n) | 0.539 | 2 | 17817 | 0.584 |
| group | 2.057 | 1 | 17817 | 0.152 |
| maintenance beta power (n) | 2.777 | 1 | 17817 | 0.096 |
| post-response beta power (n-1) | 1.783 | 1 | 17817 | 0.182 |
| set size (n-1) | 0.550 | 2 | 17817 | 0.577 |
| set size (n):group | 2.119 | 2 | 17817 | 0.120 |
| set size (n):maintenance beta power (n) | 0.615 | 2 | 17817 | 0.541 |
| group:maintenance beta power (n) | 1.934 | 1 | 17817 | 0.164 |
| set size (n):post-response beta power (n-1) | 1.428 | 2 | 17817 | 0.240 |
| group:post-response beta power (n-1) | 4.707 | 1 | 17817 | **0.030** |
| maintenance beta power (n):post-response beta power (n-1) | 2.248 | 1 | 17817 | 0.134 |
| set size (n):set size (n-1) | 0.224 | 4 | 17817 | 0.925 |
| group:set size (n-1) | 1.207 | 2 | 17817 | 0.299 |
| maintenance beta power (n):set size (n-1) | 0.694 | 2 | 17817 | 0.500 |
| post-response beta power (n-1):set size (n-1) | 3.614 | 2 | 17817 | **0.027** |
| set size (n):group:maintenance beta power (n) | 0.618 | 2 | 17817 | 0.539 |
| set size (n):group:post-response beta power (n-1) | 0.946 | 2 | 17817 | 0.388 |
| set size (n):maintenance beta power (n):post-response beta power (n-1) | 0.803 | 2 | 17817 | 0.448 |
| group:maintenance beta power (n):post-response beta power (n-1) | 4.175 | 1 | 17817 | 0.041 |
| set size (n):group:set size (n-1) | 0.999 | 4 | 17817 | 0.406 |
| set size (n):maintenance beta power (n):set size (n-1) | 0.374 | 4 | 17817 | 0.827 |
| group:maintenance beta power (n):set size (n-1) | 1.294 | 2 | 17817 | 0.274 |
| set size (n):post-response beta power (n-1):set size (n-1) | 2.806 | 4 | 17817 | **0.024** |
| group:post-response beta power (n-1):set size (n-1) | 3.135 | 2 | 17817 | **0.044** |
| maintenance beta power (n):post-response beta power (n-1):set size (n-1) | 2.514 | 2 | 17817 | 0.081 |
| set size (n):group:maintenance beta power (n):post-response beta power (n-1) | 0.806 | 2 | 17817 | 0.447 |
| set size (n):group:maintenance beta power (n):set size (n-1) | 0.865 | 4 | 17817 | 0.484 |
| set size (n):group:post-response beta power (n-1):set size (n-1) | 1.557 | 4 | 17817 | 0.183 |
| set size (n):maintenance beta power (n):post-response beta power (n-1):set size (n-1) | 1.792 | 4 | 17817 | 0.127 |
| group:maintenance beta power (n):post-response beta power (n-1):set size (n-1) | 2.639 | 2 | 17817 | 0.071 |
| set size (n):group:maintenance beta power (n):post-response beta power (n-1):set size (n-1) | 1.125 | 4 | 17817 | 0.342 |

With the three-way interaction effects (age x post-response beta power (n-1) x set size (n-1) and set size (n) x post-response beta power (n-1) x set size (n-1)), we separated the data based on set size of the previous trial (n-1) and performed a new GLMM with the formula ‘‘Accuracy (n) ~ set size (n) * maintenance beta power (n) * post-response beta power (n-1) + (1 + set size (n) + maintenance beta power (n) + post-response beta power (n-1) | participant)’. When the previous trial had four items, the age x post-response beta power (n-1) interaction effect (F(1,5972) = 3.899, p = 0.048) and set size (n) x post-response beta power (n-1) interaction effect (F(1, 5972) = 3.389, p = 0.034) were significant. Simple analysis on the modulation effect of set size of the current trial on post-response beta power did not reveal any significant results (Fig. S3A, ps > 0.126). However, as shown in Fig. S3B, when examining the age difference, stronger post-response beta power of trial n-1 would lead to a better performance of the current trial for older adults (t(2918) = 2.348, p = 0.018, Cohen’s d = 0.127) but not for younger adults (t(3074) = -0.281, p = 0.780).

| Previous trials’ set size =1 | | | | |
| --- | --- | --- | --- | --- |
| Term | FStat | DF1 | DF2 | pValue |
| (Intercept) | 29.007 | 1 | 5869 | 0.000 |
| set size (n) | 1.369 | 2 | 5869 | 0.254 |
| group | 0.913 | 1 | 5869 | 0.339 |
| maintenance beta power (n) | 1.552 | 1 | 5869 | 0.213 |
| post-response beta power (n-1) | 1.601 | 1 | 5869 | 0.206 |
| set size (n):group | 1.183 | 2 | 5869 | 0.307 |
| set size (n):maintenance beta power (n) | 0.276 | 2 | 5869 | 0.759 |
| group:maintenance beta power (n) | 1.126 | 1 | 5869 | 0.289 |
| **set size (n):post-response beta power (n-1)** | 1.362 | 2 | 5869 | 0.256 |
| **group:post-response beta power (n-1)** | 3.696 | 1 | 5869 | 0.055 |
| maintenance beta power (n):post-response beta power (n-1) | 2.157 | 1 | 5869 | 0.142 |
| set size (n):group:maintenance beta power (n) | 0.369 | 2 | 5869 | 0.692 |
| set size (n):group:post-response beta power (n-1) | 0.703 | 2 | 5869 | 0.495 |
| set size (n):maintenance beta power (n):post-response beta power (n-1) | 0.771 | 2 | 5869 | 0.463 |
| group:maintenance beta power (n):post-response beta power (n-1) | 3.903 | 1 | 5869 | 0.048 |
| set size (n):group:maintenance beta power (n):post-response beta power (n-1) | 0.777 | 2 | 5869 | 0.460 |

| Previous trials’ set size = 2 | | | | |
| --- | --- | --- | --- | --- |
| Term | FStat | DF1 | DF2 | pValue |
| (Intercept) | 44.564 | 1 | 5976 | 0.000 |
| set size (n) | 3.121 | 2 | 5976 | 0.044 |
| group | 0.019 | 1 | 5976 | 0.891 |
| maintenance beta power (n) | 0.063 | 1 | 5976 | 0.802 |
| post-response beta power (n-1) | 1.382 | 1 | 5976 | 0.240 |
| set size (n):group | 1.298 | 2 | 5976 | 0.273 |
| set size (n):maintenance beta power (n) | 0.420 | 2 | 5976 | 0.657 |
| group:maintenance beta power (n) | 0.004 | 1 | 5976 | 0.950 |
| **set size (n):post-response beta power (n-1)** | 1.311 | 2 | 5976 | 0.270 |
| **group:post-response beta power (n-1)** | 0.988 | 1 | 5976 | 0.320 |
| maintenance beta power (n):post-response beta power (n-1) | 1.460 | 1 | 5976 | 0.227 |
| set size (n):group:maintenance beta power (n) | 0.872 | 2 | 5976 | 0.418 |
| set size (n):group:post-response beta power (n-1) | 0.815 | 2 | 5976 | 0.443 |
| set size (n):maintenance beta power (n):post-response beta power (n-1) | 1.623 | 2 | 5976 | 0.197 |
| group:maintenance beta power (n):post-response beta power (n-1) | 1.628 | 1 | 5976 | 0.202 |
| set size (n):group:maintenance beta power (n):post-response beta power (n-1) | 1.194 | 2 | 5976 | 0.303 |

| Previous trials’ set size = 4 | | | | |
| --- | --- | --- | --- | --- |
| Term | FStat | DF1 | DF2 | pValue |
| (Intercept) | 50.899 | 1 | 5972 | 0.000 |
| set size (n) | 3.193 | 2 | 5972 | 0.041 |
| group | 0.196 | 1 | 5972 | 0.658 |
| maintenance beta power (n) | 0.001 | 1 | 5972 | 0.980 |
| post-response beta power (n-1) | 5.944 | 1 | 5972 | 0.015 |
| set size (n):group | 0.083 | 2 | 5972 | 0.920 |
| set size (n):maintenance beta power (n) | 0.833 | 2 | 5972 | 0.435 |
| group:maintenance beta power (n) | 0.736 | 1 | 5972 | 0.391 |
| **set size (n):post-response beta power (n-1)** | 3.389 | 2 | 5972 | **0.034** |
| **group:post-response beta power (n-1)** | 3.899 | 1 | 5972 | **0.048** |
| maintenance beta power (n):post-response beta power (n-1) | 2.376 | 1 | 5972 | 0.123 |
| set size (n):group:maintenance beta power (n) | 0.582 | 2 | 5972 | 0.559 |
| set size (n):group:post-response beta power (n-1) | 2.416 | 2 | 5972 | 0.089 |
| set size (n):maintenance beta power (n):post-response beta power (n-1) | 1.312 | 2 | 5972 | 0.269 |
| group:maintenance beta power (n):post-response beta power (n-1) | 0.592 | 1 | 5972 | 0.442 |
| set size (n):group:maintenance beta power (n):post-response beta power (n-1) | 0.835 | 2 | 5972 | 0.434 |
